# Interest and need for continuing medical education in pediatric complementary and integrative medicine: a cross-sectional survey from Switzerland

**DOI:** 10.1186/s12906-022-03581-6

**Published:** 2022-04-13

**Authors:** Benedikt M. Huber, Pierre-Yves Rodondi

**Affiliations:** 1 Center for Integrative Pediatrics, Department of Pediatrics, Fribourg Cantonal Hospital, Fribourg, Switzerland; 2 Pediatrics, Department of Community Health, Faculty of Science and Medicine, University of Fribourg, Fribourg, Switzerland; 3 Center for Integrative Pediatrics, Department of Pediatrics, HFR Fribourg – Cantonal Hospital, Chemin des Pensionnats 2-6, 1708 Fribourg, Switzerland; 4 Institute of Family Medicine, University of Fribourg, Fribourg, Switzerland

**Keywords:** Pediatric integrative medicine, Complementary medicine, Pediatrics, Switzerland, Continuing medical education, Training, Evaluation

## Abstract

**Background:**

Pediatric integrative medicine, combining conventional and complementary medical approaches for children and adolescents, is an integral part of the health care system in Switzerland. However, there is still a lack of complementary and integrative medicine topics in training and continuing educational programs. For the first time on a national level, the 2021 annual conference of the Swiss Society of Pediatrics was entirely dedicated to the topic of integrative medicine.

**Methods:**

Using a cross-sectional online survey, this study investigated congress participants’ evaluation and feedback with the aim to assess whether the program had met their objectives and to get empirical data on their attitude, expectations and needs regarding pediatric complementary and integrative medicine. Descriptive methods were used to present the results.

**Results:**

Among 632 participants of the conference, 228 completed the evaluation form (response rate 36%). The overall feedback about the congress and the main theme of pediatric integrative medicine was clearly positive. The majority of respondents had achieved their educational objectives including complementary and integrative medicine issues. 82% were motivated to learn more about complementary and integrative medicine and 66% were stimulated to integrate complementary therapies into their professional practice.

**Conclusion:**

This study from Switzerland confirms the interest in integrative medicine among pediatricians and supports the need for pre- and postgraduate pediatric training on topics related to complementary and integrative medicine. Developing and adapting training and continuing medical education based on evaluations of participant feedback can promote professional development and improve patient care for the benefit of physicians and patients.

**Supplementary Information:**

The online version contains supplementary material available at 10.1186/s12906-022-03581-6.

## Background

Pediatric integrative medicine (PIM) describes a patient- and family-centered approach using all appropriate conventional and complementary therapies, health care professionals and disciplines based on scientific evidence and clinical experience, to promote health and healing for children and adolescents [1–3]. In Switzerland, which is known for its high standards in child health care [4], PIM is an integral part of the health care system [3]. This is shown, on the one hand, by the significant prevalence of complementary medicine use within the Swiss population in general [5–7] and among children and adolescents in particular, concerning the whole spectrum from primary care to highly specialized medicine [8–13]. According to data from the federal office of statistics, the utilization of complementary medicine is constantly increasing during the last two decades [14]. On the other hand, there are pediatricians and family physicians offering an integrative medicine approach to their patients based on a double training in conventional and complementary medicine [13, 15]. In recent years, complementary and integrative medicine (C&IM) is attracting a growing number of physicians looking for a more holistic and health orientated focus for their practice and for an expansion of therapeutic options. A national survey among pediatricians confirmed this observation, with 63% of respondents expressing their interest in courses and trainings on C&IM [13].

In contrast, C&IM topics are still hardly found on the agenda of pediatric training and continuing medical education (CME) events in Switzerland. This is particularly relevant since almost all pediatricians are regularly confronted with questions related to C&IM [13]. Even if some pediatricians would choose a complete specialist training in complementary medicine, some basic knowledge is essential to provide competent counseling on safety and efficacy of complementary therapies. Lack of knowledge and understanding of patients concerns is one important reason driving families away from pediatricians to medical and non-medical providers of complementary medicine [16]. As stated by Ring et al., “integrative medicine CME activities are needed to bridge the knowledge gap in service of both these populations: patients who want appropriate integrative options and medical doctors who seek to enhance their practice” [17].

The growing importance and the strong interest expressed by pediatricians [13] gave reason, to choose PIM as the main theme for the 2021 annual conference of the Swiss Society of Pediatrics (SSP). This conference traditionally plays an important role in pediatric CME in Switzerland. While single lectures or sessions on topics related to C&IM have already been part of previous pediatric conferences in various countries, this was, to our knowledge, the first national pediatric congress worldwide entirely dedicated to integrative medicine. It must be emphasized, that the congress was not limited to complementary medicine topics in pediatrics. Just as integrative medicine includes the whole spectrum of conventional and complementary medical approaches, the scientific program covered various topics from conventional and complementary medicine in a balanced way to strengthen the vision of integrative medicine. An additional file shows the program at a glance with all topics [see Additional file 1].

Originally planned for 2020, this conference had to be postponed due to the Covid-19 pandemic and was finally transformed to a virtual event via an online platform. The virtual mode was completely novel for conferences of the SSP and brought advantages (e.g. participation from everywhere without travelling) and disadvantages (e.g. technical challenges, lack of direct interaction, personal contact and social exchange). The SSP, the Swiss Society of General Internal Medicine and the European Accreditation Council for Continuing Medical Education (EACCME) accredited the congress as live educational event.

Because integrative medicine is an innovative and sometimes controversial theme [17, 18], and because this was the first national conference on PIM organized in response to the request of courses and trainings on C&IM [13], a systematic analysis of attendees’ evaluation and feedback was indicated. Based on the results of our previous survey [13], we assumed that the overall feedback on the congress and the main theme of PIM would be positive. The aim of the study was to assess this feedback and whether or not the program had met their objectives. An additional objective was to get empirical data on participants’ attitudes, expectations and needs regarding C&IM in pediatrics. These could give directions for the planning of future CME activities.

## Material and methods

We conducted a cross-sectional survey with participants of the 2021 annual conference of the SSP. The virtual mode facilitated both the evaluation for participants and the subsequent data analysis. An online questionnaire was available during the congress and afterwards for a period of 2 weeks. Accessibility was linked to the personal login to the platform. All participants of the event were eligible for the study. They were actively invited to take part in the survey (through pop-ups on the event platform) and participation was voluntary. The target audiences of the SSP conferences are pediatricians, general practitioners and other medical doctors treating children and adolescents (e.g. school doctors, child surgeons, child and adolescent psychiatrists, etc.). Included are physicians in training as well as researchers in the above-mentioned fields. For the 2021 congress, the target audience expanded to international participants, as the virtual event was accessible online without travelling.

We defined the general attitude (feedback) of the participants as primary outcome of our analysis. This was assessed with different questions regarding personal benefit from the participation, quality of speakers/faculty and scientific content and whether the participant would recommend this event to others. As secondary outcomes, we defined the meeting of predefined educational objectives with a focus on C&IM issues and the C&IM topics suggested for future events. We expected that a first national PIM conference would further raise awareness of this subject and would thus stimulate participants to express their specific interests and needs regarding C&IM. We therefore searched the answers to the question asking participants for any clinical issues or problems within their scope of practice they would like to see addressed in future conferences and extracted specific C&IM theme requests or suggestions.

A questionnaire (evaluation form) was created based on literature on CME evaluation [19–21] and according to standards of the EACCME [22], also using examples of different previously accredited CME events. Questions were selected and adapted to the Swiss context. We added specific questions on the subject of C&IM taking into account our previous research [13]. Overall, the questionnaire consisted of almost 60 items organized in three sections giving the opportunity for detailed feedback and analysis. The first section covered questions requiring feedback about the overall congress and the individual lectures, sessions and workshops participants attended. Additional questions were related to CME / continuing professional development assessing whether educational objectives were met. A special focus was placed on attitudes, expectations and needs regarding C&IM. The second and third section concerned questions on organizational and marketing issues as well as the evaluation of the online platform and the virtual exhibition area. They were not included in this analysis, as they were only relevant for the organization team.

The questionnaire was constructed and made available in English. It was estimated to take about 5 min to answer the entire survey. In the analyzed first section of the questionnaire, most questions and items were closed-ended using a five-point Likert scale on which respondents could indicate their level of agreement (strongly agree – agree – neutral – disagree – strongly disagree). Open-ended questions were used to inquire about topics that participants would like to see addressed in future events and to get feedback on what they liked or what should be improved. If the respondent could not answer a question or was not concerned by a question, he/she could reply “not applicable”.

All data was completely anonymized for the analysis. Data on the total number of congress participants as well as their country of origin, recorded at the registration, were provided by the congress organization and could not be linked to the survey data. The study was carried out in accordance with relevant guidelines and regulations and the cantonal ethics commission confirmed that the study was outside the scope of the Swiss Federal Human Research Act.

Survey data were imported in Microsoft Excel. Descriptive statistics were used to summarize the responses and to present the results. We calculated the overall response rate (surveys completed / participants of the congress) and for accurate evaluation the response rates for each item (answers to each question / participants of the congress). Missing answers or abstentions were excluded from the analysis.

## Results

Overall, 632 participants attended the 2021 SSP conference including faculty members (speakers, chairs). 596 (94%) came from Switzerland, 36 (6%) were from abroad (various European countries 30, USA 2, Canada 2, Brazil 1, Singapore 1). As the medical specialties were not recorded at the registration nor asked in the survey, we can only assume based on previous experiences, that the vast majority of participants were board certified pediatricians or pediatricians in training.

A total of 228 participants completed the survey (response rate 36%). However, the response rate of individual questions could be lower due to abstentions or incomplete response to the survey. The latter mainly concerned the questions on organization and marketing, which were not considered for this study.

The overall feedback and general attitude about the congress and the main theme of PIM was clearly positive (Table 1). The majority of responding attendees agreed or strongly agreed that this conference improved their competencies and performance and would change their practice. 82% were motivated to learn more about C&IM. Additionally, the largest part of respondents agreed or strongly agreed that the quality of speakers was excellent and that the quality and evidence base of the scientific content was high. Finally, 80% would recommend this congress to others.

**Table 1:** Evaluation of the 2021 Swiss Society of Pediatrics congress by participants

Item (n, response rate)	Strongly Agree	Agree	Neutral	Disagree	Strongly Disagree	N/A
This educational event improved my professional competencies (*n* = 228, 36%)	35 (15%)	161 (71%)	29 (13%)	1 (0.4%)	0 (0%)	2 (0.9%)
Participating in this educational event will improve my professional performance (*n* = 228, 36%)	27 (12%)	146 (64%)	50 (22%)	1 (0.4%)	0 (0%)	4 (2%)
I will make changes to my professional practice based on what I learned (*n* = 224, 35%)	20 (9%)	147 (66%)	50 (22%)	3 (1%)	0 (0%)	4 (2%)
I was motivated to learn more about complementary and integrative medicine (*n* = 228, 36%)	55 (24%)	133 (58%)	30 (13%)	8 (4%)	0 (0%)	2 (0.9%)
I was stimulated to integrate complementary therapies into my professional practice (*n* = 228, 36%)	35 (15%)	116 (51%)	63 (28%)	10 (4%)	1 (0.4%)	3 (1%)
Overall, the virtual educational event met my expectations (*n* = 227, 36%)	39 (17%)	134 (59%)	45 (20%)	5 (2%)	1 (0.4%)	3 (1%)
After attending the event, I am able to address individual needs in compliance with my continuing professional development plan (*n* = 140, 22%)	12 (9%)	72 (51%)	40 (29%)	3 (2%)	1 (0.7%)	12 (9%)
The overall quality of the speakers / faculty was excellent (*n* = 227, 36%)	49 (22%)	150 (66%)	23 (10%)	3 (1%)	0 (0%)	2 (0.9%)
The scientific / educational content was of high quality (*n* = 224, 35%)	41 (18%)	142 (62%)	33 (14%)	4 (2%)	0 (0%)	4 (2%)
The scientific / educational content was evidence based (*n* = 225, 36%)	30 (13%)	126 (56%)	59 (26%)	3 (1%)	1 (0.4%)	6 (3%)
The accredited content was balanced, objective, and free from commercial bias (*n* = 227, 36%)	40 (18%)	137 (60%)	41 (18%)	1 (0.4%)	0 (0%)	8 (4%)
I would recommend this educational event to others (*n* = 225, 36%)	44 (20%)	136 (60%)	38 (17%)	1 (0.4%)	2 (0.9%)	4 (2%)

*Abbreviation*: *N/A* Not applicable. Note: Percentages may add to +/− 100 due to rounding

The response rates to the questions about educational objectives were each 22%. The results concerning educational objectives related specifically to C&IM are presented in Table 2. Overall, respondents indicated they had achieved predefined educational objectives, however to varying degrees. While 84% of respondents agreed or strongly agreed to be able to describe the concept of integrative medicine and its realization in pediatrics, the agreement was less strong for the ability to establish a personalized training program in complementary medicine, to network with international PIM experts or to discuss potentials and challenges of PIM research, with agreement or strong agreement in only 31, 40 and 51%, respectively. 66% agreed or strongly agreed that they have gained knowledge of complementary and integrative medicine that enables them to have open communication with patients and families about the use of all treatment modalities.

**Table 2:** Responses of participants of the 2021 Swiss Society of Pediatrics congress concerning educational objectives

After attending the event, you are able to ... (n, response rate)	Strongly Agree	Agree	Neutral	Disagree	Strongly Disagree	N/A
Explore and discuss new approaches for current challenges in medicine (*n* = 141, 22%)	21 (15%)	84 (60%)	26 (18%)	4 (3%)	0 (0%)	6 (4%)
Describe the concept of integrative medicine and its realization in pediatrics (*n* = 140, 22%)	33 (24%)	85 (61%)	17 (12%)	1 (0.7%)	0 (0%)	4 (3%)
Identify different types of complementary medicine most frequently used and provided in pediatrics (*n* = 142, 22%)	31 (22%)	87 (61%)	18 (13%)	3 (2%)	0 (0%)	3 (2%)
Establish a personalized training program in complementary medicine based on information provided during the congress (*n* = 141, 22%)	7 (5%)	37 (26%)	69 (49%)	14 (10%)	2 (1%)	12 (9%)
Evaluate indications and contraindications of specific complementary therapies for children and adolescents (*n* = 142, 22%)	12 (8%)	74 (52%)	39 (27%)	5 (4%)	0 (0%)	12 (8%)
Define opportunities and limitations for the use of complementary therapies in children and adolescents (*n* = 141, 22%)	10 (7%)	75 (53%)	39 (27%)	4 (3%)	1 (0.7%)	12 (9%)
Conduct an open communication with patients and families on the use of all treatments based on acquired knowledge on complementary and integrative medicine (*n* = 141, 22%)	20 (14%)	74 (52%)	36 (25%)	6 (4%)	0 (0%)	5 (4%)
Network with international experts of pediatric integrative medicine fostering collaboration and research in the field (*n* = 140, 22%)	17 (12%)	40 (28%)	53 (37%)	9 (6%)	1 (0.7%)	20 (14%)
Assess safety issues of conventional and complementary therapies (*n* = 141, 22%)	15 (11%)	77 (55%)	42 (30%)	2 (1%)	1 (0.7%)	4 (3%)
Discuss potentials and challenges of research in pediatric integrative medicine (*n* = 140, 22%)	7 (5%)	64 (46%)	50 (36%)	8 (6%)	1 (0.7%)	10 (7%)

*Abbreviation*: N/A Not applicable. Note: Percentages may add to +/− 100 due to rounding

A total of 70 participants (response rate 11%) suggested topics for future conferences. These covered a great variety of themes, therapeutic approaches and diseases/problems from the perinatal period to adolescence. Table 3 shows the suggestions related specifically to C&IM that attendees would like to see addressed in future pediatric conferences.

**Table 3:** Complementary and integrative medicine topics attendees suggested for future pediatric conferences

• Continuation of the integrative approach	
• More integrative medicine	
• Concrete addresses for more educational events like this and for institutions maybe of local experts who would like to share their knowledge	
• Addresses of known and recommendable institutions	
• Integrative medicine and time management	
• Consider cultural aspects in the context of integrative medicine	
• Intercultural medicine aspects, culture specific approaches in communication and therapies, also in the context of integrative medicine	
• General updates, even for integrative pediatrics	
• How to apply complementary medicine in the emergency department, with specific examples and protocols, and with references	
• Clinical applications of the suggestions/all day life applications of complementary medicine	
• More practical and specific information and skills for general pediatricians to use complementary medicine, without having to specialize in the field	
• More practice-based presentations with tips and recommendations for everyday practice	
• A conference of the SSP focusing more on practical issues of the life in a pediatric practice	
• Concrete complementary therapeutic strategies for example in recurrent acute otitis media	
• Follow up of the effects of complementary medicine	
• Use of hypnosis therapy in pediatrics	
• Phytotherapy (herbal medicine)	
• Homeopathy	
• Frequent skin problems and practical tools. In general, the topics covered are good but I find that the practical side for the daily activity is missing.	
• Recurrent *Enterobius vermicularis* infections which complementary treatments	
• Postpartum depression in mothers, complementary medicine as a possible help	

## Discussion

Analyzing the evaluation of the 2021 national pediatric conference in Switzerland on the topic of PIM, this study clearly confirms a strong interest and need for continuing education and training in pediatric C&IM. In support of our main assumption, the results reveal a very positive general feedback on the congress and an appreciation of the main theme. By far the largest part of the respondents indicated a personal benefit from the participation in terms of professional competencies, performance and practice. In addition, attendees judged the quality of the speakers and the scientific content to be high. Especially the positive feedback on issues related to C&IM confirms the findings of the 2017 national survey among pediatricians on complementary medicine [13] and justify the choice of the main theme for this large pediatric CME event in Switzerland. Furthermore, our results show that the congress could raise awareness of C&IM and was able to provide knowledge that is relevant for daily practice. For example, a majority stated that they were encouraged to communicate openly with patients about all treatment options as a result of the knowledge they had gained about C&IM. This is of particular importance as there is still a lack of communication between patient and physician on C&IM [12, 16]. Physicians should inform the child and his parents about potential risks and benefits of complementary therapies [23].

Concerning secondary outcomes, the study shows that most responding participants achieved educational objectives. This was particularly the case for the objectives related to C&IM, showing that the scientific program met the interest of the participants and covered topics relevant to their scope of practice. It is not surprising, that the objectives requiring more direct exchange and networking were less easily achieved due to the conditions of the congress as a virtual event. Against our expectations, only very few participants used the opportunity to suggest topics for future conferences. This also concerned the topics specifically related to C&IM. These were, however, clearly in favor of continuing education and training in C&IM. Respondents repeatedly requested the teaching of practical recommendations for complementary therapies, which confirms the search for expanding therapeutic options. Although not directly linked, many other suggested topics like communication or interdisciplinarity play also a central role in the concept of integrative medicine [2, 16, 24]. Finally, we can only speculate, if the respondents had the PIM approach in mind when suggesting a large number of medical disorders and diseases from asthma and allergy to autism, from functional disorders to pediatric surgery, or from diseases of the newborn to adolescent medicine. As PIM is not a pediatric subspecialty but rather a general approach that can be applied to any medical problem [2, 25], it would be possible to discuss all these topics also from the perspective of PIM and to evaluate whether or not they could benefit from an integrative medicine approach. Demonstrating evidence of an added value of integrative medicine compared to the conventional approach alone is the major task that lies ahead. Research in PIM is still at the outset of its development [26] and Switzerland would offer optimal conditions based on a long tradition and an excellent infrastructure for pediatric research [4, 27].

The total number of participants was within the range of SSP conferences of previous years. However, it seems impossible to draw a conclusion from this for the chosen topic, as the circumstances of the event due to the Covid-19 pandemic and the virtual mode may have influenced the number of participants more than the content of the program. In contrast to our expectations, the conference attracted only very few participants from abroad despite an easy accessibility via the online platform.

This CME evaluation study has several limitations. First, the lack of demographic data of respondents prevents further analysis and a generalization of the results. While the collection of demographic data is not universally done and recommended for CME evaluations [19], this could be of interest if the participant feedback is to be used for the planning of tailored continuing educational events. Second, a response rate of 36% reflects only a part of the participants. Additionally, a preselection of those pediatricians being particularly interested in the topic of the congress could at least partially explain the positive results in favor of C&IM within the sample of respondents. However, this applies to any subject of a scientific congress and does not question the choice of the topic. Third, the study used a cross-sectional design and could therefore not assess a change in knowledge or clinical practice [21]. It cannot be answered whether or not the congress actually had an impact on the professional performance and improved patient care. Finally, a participant could respond the survey more than once. However, it seems unlikely that multiple responses were used to deliberately influence the evaluation.

Unfortunately, there are no similar CME evaluation studies from pediatrics or pediatric C&IM, which would allow a comparison and contextualization of our results. This study might serve as a model and we want to encourage CME organizers not only to include C&IM topics in their programs but also to systematically collect, analyze and publish the participants’ evaluations. This could certainly help and guide the development of future CME activities resulting in improved educational programs with an impact on continuing professional development and patient care.

## Conclusion

PIM is an innovative concept with increasing importance and global dissemination. This study from Switzerland confirms the interest in PIM among pediatricians and supports the need for pre- and postgraduate pediatric training on topics related to C&IM. Developing and adapting such training and CME events based on evaluations of participant feedback can promote professional development and improve patient care for the benefit of physicians and patients.

## Supplementary Information

**Additional file 1.**

## Data Availability

All data generated and analyzed during this study are included in this published article.

## Abbreviations

C&IM: Complementary and Integrative Medicine
CME: Continuing Medical Education
EACCME: European Accreditation Council for Continuing Medical Education
N/A: Not applicable
PIM: Pediatric Integrative Medicine
SSP: Swiss Society of Pediatrics

## References

[1] Academic consortium for integrative medicine and health. Definition of integrative medicine and health. Available from: https://imconsortium.org/about/introduction/. Accessed 25 Oct 2021.

[2] McClafferty H, Vohra S, Bailey M (2017). Pediatric integrative medicine. Pediatrics..

[3] Huber BM, Rodondi PY, Wildhaber J (2020). La pédiatrie intégrative fait partie intégrante des soins pédiatriques en Suisse. Rev Med Suisse.

[4] Jenni OG, Sennhauser FH (2016). Child health care in Switzerland. J Pediatr.

[5] Wolf U, Maxion-Bergemann S, Bornhöft G, Matthiessen PF, Wolf M (2006). Use of complementary medicine in Switzerland. Forsch Komplementärmed.

[6] Klein SD, Frei-Erb M, Wolf U (2012). Usage of complementary medicine across Switzerland. Swiss Med Wkly.

[7] Klein SD, Torchetti L, Frei-Erb M, Wolf U (2015). Usage of complementary medicine in Switzerland: results oft he Swiss Health Survey 2012 and development since 2007. PLoS One.

[8] Moenkhoff M, Baenziger O, Fischer J, Fanconi S (1999). Parental attitude towards alternative medicine in the paediatric intensive care unit. Eur J Pediatr.

[9] Zuzak TJ, Zuzak-Siegrist I, Simoes-Wüst AP, Rist L, Staubli G (2009). Use of complementary and alternative medicine by patients presenting to a paediatric emergency department. Eur J Pediatr.

[10] Zuzak TJ, Zuzak-Siegrist I, Rist L, Staubli G, Simoes-Wüst AP (2010). Medicinal systems of complementary and alternative medicine: a cross-sectional survey at a pediatric emergency department. J Altern Complement Med.

[11] Magi T, Kuehni CE, Torchetti L, Wengenroth L, Luer S, Frei-Erb M (2015). Use of complementary and alternative medicine in children with cancer: a study at a Swiss University hospital. PLoS One.

[12] Lüthi E, Diezi M, Danon N, Dubois J, Pasquier J, Burnand B, Rodondi PY (2021). Complementary and alternative medicine use by pediatric oncology patients before, during, and after treatment. BMC Complement Med Ther.

[13] Huber BM, von Schoen-Angerer T, Hasselmann O, Wildhaber J, Wolf U (2019). Swiss paediatrician survey on complementary medicine. Swiss Med Wkly.

[14] Federal Office of Statistics. Inanspruchnahme von Komplementärmedizin in den letzten 12 Monaten. Available from: https://www.bfs.admin.ch/bfs/de/home/statistiken/kataloge-datenbanken/tabellen.assetdetail.7586143.html. Accessed 25 Oct 2021.

[15] Déglon-Fischer A, Barth J, Ausfeld-Hafter B (2009). Komplementärmedizin in Schweizer Praxen der Grundversorgung [Complementary and alternative medicine in primary care in Switzerland]. Forsch Komplementmed.

[16] Foley H, Steel A, Cramer H, Wardle J, Adams J (2019). Disclosure of complementary medicine use to medical providers: a systematic review and meta-analysis. Sci Rep.

[17] Ring M, Majd I, Mehta DH (2020). Keeping integrative medicine continuing medical education on the cutting edge – and compliant. J Altern Complement Med.

[18] Sierpina VS, Kreitzer MJ (2010). The continuing bias against complementary and integrative healthcare education. Explore..

[19] Coldeway NA, DeLisa JA (1986). Evaluation of continuing medical education: making a difference. Arch Phys Med Rehabil.

[20] Shannon S (2003). Programme evaluation in CME. Lancet..

[21] Tian J, Atkinson NL, Portnoy B, Gold RS (2007). Systematic review of evaluation in formal continuing medical education. J Contin Educ Heal Prof.

[22] Union Européenne des Médecins Spécialistes (UEMS), European Accreditation Council for Continuing Medical Education (EACCME). EACCME criteria for the accreditation of live educational events (LEE). UEMS 2016.20. Available from: https://eaccme.uems.eu/accreditationlee.aspx. Accessed 25 Oct 2021.

[23] Gilmour J, Harrison C, Asadi L, Cohen MH, Vohra S (2011). Informed consent: advising patients and parents about complementary and alternative medicine therapies. Pediatrics.

[24] Sibinga EM, Duggan OMC, AK, Wilson MH. (2004). Parent-pediatrician communication about complementary and alternative medicine use for children. Clin Pediatr (Phila).

[25] Esparham A, Misra SM, Sibinga E, Culbert T, Kemper K (2018). Pediatric integrative medicine: vision for the future. Children (Basel).

[26] Vohra S, Zorzela L, Kemper K, Vlieger A, Pintov S (2019). Setting a research agenda for pediatric complementary and integrative medicine: a consensus approach. Complement Ther Med.

[27] SwissPedNet – the Swiss research network of clinical pediatric hubs. Available from: https://www.swisspednet.ch/home. Accessed 25 Oct 2021.

