# Supplementary material for: Interest and need for continuing medical education in pediatric complementary and integrative medicine: a cross-sectional survey from Switzerland
Source: BMC Complement Med Ther. 2022 Apr 13;22:106. doi: 10.1186/s12906-022-03581-6 (PMC9007250; doi:10.1186/s12906-022-03581-6)
Supplement: Supplementary file 1 — Additional file 1. [file 12906_2022_3581_MOESM1_ESM.pdf]

PROGRAM AT A GLANCE – Pédiatrie Suisse Virtual Congres 2021 **Donnerstag | Jeudi | Thursday, 10.6.2021**

	Room 1	Room 2	Room 3	Room 4	Room 5	Room 6
07:30	Opening of online registration					
08:00 – 09:15	**Plenary 1** Integrative Pediatrics					
09:15 – 09:30	Networking Break (coffee | view ePoster | visit exhibition | chat)					
09:30 – 10:30	**Session 1** Infections, antibiotics and complementary medicine	**Session 2** Pediatric immunology	**Session 3** Chronic pain (integrative & inter-disciplinary approach)	**Workshop 1** Self-Awareness/ Self-Care for physicians	**SwissPedNet** Research Session 1	**Oral presentations 1**
10:30 – 11:00	Networking Break (coffee | view ePoster | visit exhibition | chat)					
11:00 – 12:00	**Plenary 2** Psychosomatics & Eating disorders					
12:00 – 13:00	Networking Break (lunch | view ePoster | visit exhibition | chat)					
13:00 – 14:00	**Session 4** Choosing wisely in pediatrics	**Session 5** Psychosomatics: abdominal pain and enuresis (German)	**Session 6** Psychosomatics: abdominal pain and enuresis (French)		**SwissPedNet** Research Session 2	**Oral presentations 2**
14:00 – 14:15	Networking Break (coffee | view ePoster | visit exhibition | chat)					
14:15 – 15:45	**Session 7** Integrative medicine in children's hospitals	**Session 8** Update on vaccination		**Session 9** Integrative pediatric oncology	**Workshop 2** Mindfulness (French)	**Workshop 3** Mindfulness (German)
15:45 – 16:15	Networking Break (coffee | view ePoster | visit exhibition | chat)					
16:15 – 17:15	**Session 10** COVID-19 News and Views	**Session 11** Child abuse (German)	**Session 12** Child abuse (French)		**Workshop 4** Motivational interview (German)	**Workshop 5** Motivational interview (French)
17:15 – 17:30	Networking Break (coffee | view ePoster | visit exhibition | chat)					
17:30 – 18:30	**Plenary 3** Pediatric integrative medicine (global) & Integrative treatment of ADHD					
18.30 – 18.45	Networking Break (apéro | view ePoster | visit exhibition | chat)					
18:45 – 19:45	**Meet the Expert 1** Shared decision making	**Meet the Expert 2** Homeopathy (German)	**Meet the Expert 3** Homeopathy (French)	**Meet the Expert 4** Integrative approach to ADHD & Autism		SIGIP meets PIM Leaders (on invitation only)
19.45	End of first congress day					

PROGRAM AT A GLANCE – Pédiatrie Suisse Virtual Congres 2021

Freitag | Vendredi | Friday, 11.6.2021

	Room 1	Room 2	Room 3	Room 4	Room 5	Room 6
07:30	Opening of online registration					
08:00 – 09:00	**Meet the Expert 5** Phytotherapy for acute infections (German)	**Meet the Expert 6** Phytotherapy for acute infections (French)	**Meet the Expert 7** Integrative pediatric dermatology (German)			
09:00 – 09:15	Networking Break (coffee | view ePoster | visit exhibition | chat)					
09:15 – 10:30	**Plenary 4** Integrative approach to asthma & Adolescents and screens					
10:30 – 11:00	Networking Break (coffee | view ePoster | visit exhibition | chat)					
11:00 – 12:00	**Session 13** Digital Media	**Symposium Kinderärzte Schweiz** Complementary and integrative medicine in practice	**Meet the Expert 8** Integrative pediatric dermatology (French)	**Workshop 6** Self-Awareness/ Self-Care for physicians	**SwissPedNet** Research Session 3	**Oral presentations 3**
12:00 – 14:00	Networking Break (lunch | view ePoster | visit exhibition | chat)					
12:10 – 14:00	General assembly Swiss Society of Pediatrics					
14:00 – 15:30	**Research Session** Research in pediatric integrative medicine	**Session 14** Nutrition	**Session 15** Adolescent health	**Workshop 7** External applications (basics and practice)	**Workshop 8** Acupuncture	
15:30 – 16:00	Networking Break (coffee | view ePoster | visit exhibition | chat)					
16:00 – 17:30	**Plenary 5** Irritable bowel syndrome					
17:30 – 17:45	Networking Break (coffee | view ePoster | visit exhibition | chat)					
17:45 – 18:45	**Session 16** Fever	**Session 17** Anthroposophic medicine & Heart based medicine		**Session 18** Complementary therapies for functional abdominal pain		
18:45	End of congress					
